# The Role of DNA Insertions in Phenotypic Differentiation between Humans and Other Primates

**DOI:** 10.1093/gbe/evv012

**Published:** 2015-01-28

**Authors:** Elizabeth H.B. Hellen, Andrew D. Kern

**Affiliations:** Department of Genetics, Nelson Biolabs, Piscataway, NJ, USA

**Keywords:** indel, ape, neural, dental

## Abstract

What makes us human is one of the most interesting and enduring questions in evolutionary biology. To assist in answering this question, we have identified insertions in the human genome which cannot be found in five comparison primate species: Chimpanzee, gorilla, orangutan, gibbon, and macaque. A total of 21,269 nonpolymorphic human-specific insertions were identified, of which only 372 were found in exons. Any function conferred by the remaining 20,897 is likely to be regulatory. Many of these insertions are likely to have been fitness neutral; however, a small number has been identified in genes showing signs of positive selection. Insertions found within positively selected genes show associations to neural phenotypes, which were also enriched in the whole data set. Other phenotypes that are found to be enriched in the data set include dental and sensory perception-related phenotypes, features which are known to differ between humans and other apes. The analysis provides several likely candidates, either genes or regulatory regions, which may be involved in the processes that differentiate humans from other apes.

## Introduction

Humans and chimpanzees are estimated to have diverged from their most recent common ancestor approximately 6 million years ago (Hara et al. 2012; Scally et al. 2012). During this time, significant phenotypic differences have evolved between the two species. Differences in brain shape and size (Semendeferi and Damasio 2000), skull shape (Penin et al. 2002; Neubauer et al. 2010), skeletal and musculature differentiation (Kikuchi 2010), and changes in dentition (Fukase 2012) and digestion (Babbitt et al. 2010; Watkins et al. 2010) have all occurred. The most important differences, from a human point of view, may be the development of language, emotion, and complex ideas (Vallender 2011; Heyes 2012).

In the years since the human genome was successfully sequenced (Lander et al. 2001), a large number of other primate sequencing projects have been undertaken (Chimpanzee Sequencing and Analysis Consortium 2005; Locke et al. 2011; Scally et al. 2012). The completion of these projects has allowed for a detailed comparison between species at the DNA level. During the initial analysis of the chimpanzee genome, approximately 35 million nucleotide substitutions and 5 million indels were identified as differing between the human and chimpanzee genomes (Chimpanzee Sequencing and Analysis Consortium 2005). Previous analysis of the differences between these species, using chromosome 22, shows that a high percentage (83% in chromosome 22) of coding sequences contain differences between the two species and that 1.4% of the chromosome consisted of single base changes (Chimpanzee Sequencing and Analysis Consortium 2004). However, many of these differences are found in noncoding regions and as such do not translate to easily identifiable functional differences. While this is so, comparative genomic analysis identified sets of human accelerated regions, which show high levels of conservation in vertebrates, including nonhuman primates, but recent rapid divergence in the human genome (Pollard et al. 2006; Prabhakar et al. 2006). These regions were found to occur mostly in noncoding DNA, often close to genes involved in transcription and DNA binding, implying that these human-specific mutations might be the initiator of a cascade of changes in gene expression. These findings square well with the King and Wilson (1975) hypothesis that many phenotype-altering differences between humans and chimpanzees are related to differences in expression levels, rather than changes in coding sequence.

Although much of the variation between species is composed of single nucleotide substitutions, a substantial portion consists of indels, sequences either inserted into, or deleted from, a species or lineage. A recent study into human-specific deletions, using a comparison with the chimpanzee and macaque genomes, showed an enrichment of human-specific deletions in regions near genes associated with steroid hormone receptor activity. Deletions were also associated with genes showing expression in neural and brain-related tissues (McLean et al. 2011). A further analysis of indels found in humans, but not in other primates, showed that genes with indels found in either the coding sequence or putative regulatory regions were significantly more likely to be differentially expressed between humans and chimpanzees than were genes where indels were not found in these regions (Polavarapu et al. 2011). Together these studies suggest that recent insertion or deletion events are likely to account for some of the differences between humans and nonhuman primates.

Many of the comparisons carried out between humans and chimpanzees have concentrated on sequences deleted in the human genome in comparison with the ancestral state. However, human-specific deletions are thought to be more deleterious than insertions and more likely to be eliminated through purifying selection (Sjödin et al. 2010). An insertion event may be less likely to be subject to purifying selection than a deletion if it retains the original function while adding novel functionality through the insertion of new regulatory or functional motifs. Transposable elements (TEs) in particular are able to quickly change the regulatory landscape of a gene, through the addition of new motifs (Thornburg et al. 2006; Rebollo et al. 2012). TEs have been shown to contain motifs which can be co-opted by the host genome as transcription factor binding sites (Polavarapu et al. 2008; Emera and Wagner 2012; Testori et al. 2012), polyadenylation sites (Chen et al. 2009), or other regulatory sequences (Rebollo et al. 2012). Many of the insertions occurring in a genome are likely to be due to TEs, although other mechanisms can be responsible, such as an increase in the size of repetitive regions.

In a small number of cases, insertions have been shown to allow new protein-coding genes to arise from noncoding DNA. The human-specific gene *DNAH10OS* in particular appears to rely on a human-specific insertion to allow for correct functioning. A 10-bp human-specific insertion is found in an *DNAH10OS* exon, without which a frameshift would occur, causing early termination of the protein (Knowles and McLysaght 2009). Although the study clearly shows the capacity of insertions to create protein-coding level changes specific to the human genome, the expected number of de novo genes is low. The expectation is still that we will find a far greater number of insertions affecting regulatory changes rather than protein-coding changes. Here, we explore the extent to which human-specific insertions have arisen since the most recent common ancestor with chimpanzees by comparison of five primate species with human. Many of the insertions identified are found to be associated with phenotypic differences between humans and other apes, and as such may have contributed to the development of human-specific characteristics.

## Methods

### Data Set

Multiple alignment files for the 100 vertebrate alignment were retrieved from UCSC (Karolchik et al. 2014) and parsed to create alignments containing human (hg19), chimp (panTro4), gorilla (gorGor3), orangutan (ponAbe2), gibbon (nomLeu3), and macaque (rheMac3) sequences. The resulting primate alignment was searched for regions which contained insertions specific to the human sequence. Only human sequences with explicit matches to gap characters or annotations in each of the other species were included in an effort to reduce false positives resulting from unmapped regions in lower quality genomes. Insertions <10 nucleotides long were removed from the data set, insertions shorter than this were assumed to be less likely to contain new regulatory motifs (Bilu and Barkai 2005), and given the difficulty in accurate identification of the correct positions of short indels and therefore the increased difficulty in ensuring correct alignments, it was decided that the study would concentrate only on midlength and long insertions. The set of human-specific insertions were compared with the 1000 genomes project indel data set. Exact insertions which were found to be polymorphic in the human population were removed. Insertions containing polymorphic single nucleotide polymorphisms (SNPs) or smaller internal indels, indicative of later mutational events, were not removed.

### Identification of Related Phenotypes and Regulatory Potential

The 21,269 insertions longer than 10 nucleotides, fixed in the human population, were analyzed in a number of different ways to search for enriched phenotypes. The UCSC genome browser was used to find insertions which intersected with the set of UCSC genes including intron, exon, 3′ and 5′ regions. Genes containing insertions were analyzed using DAVID (Dennis et al. 2003) to find enriched annotations, particularly biological process gene ontology terms and tissue expression annotations. The analysis was repeated using only the set of insertions found within regions annotated as exons in the set of UCSC genes.

The full fixed insertion data set, consisting of 21,269 insertions, was analyzed using GREAT (McLean et al. 2010) with default settings and the Significant by Region Binomial View. A second set of insertions which were most likely to be involved in gene expression was created by intersecting the data with the UCSC DNase Clusters V1 (DNaseI hypersensitivity clusters in 125 cell types from ENCODE) and the transcription factor ChIP V3 tracks in the UCSC genome browser (Encode Project Consortium 2010). The resulting insertions were analyzed with GREAT using the basal plus extension gene association rules, reducing the distal value to 5 kb to find elements likely to reside within the promoter region.

The human-specific insertions were compared with the RepeatMasker track in the UCSC database which contains annotations for DNA repeat motifs such as TEs. Insertions with any overlap to RepeatMasker elements annotated in the human genome were collated in a separate data set. Groups of insertions overlapping each class of RepeatMasker element were analyzed using the default settings in GREAT. Expected frequencies of each RepeatMasker element in the set of human-specific insertions were calculated using the proportions of each element annotated in the RepeatMasker track of the human genome.

### Insertions Associated with Regions Under Positive Selection

The 1000 Genomes Selection Browser v1.0 (Pybus et al. 2014) was used to find insertions in areas showing evidence of recent positive selection. The CLR (Nielson et al. 2005) data set and the FusF (Fu 1997) data set were downloaded for each of the three populations available: CEU (Utah residents with ancestry from northern and western Europe), CHB (Han Chinese in Beijing, China), and YRI (Yoruba in Ibadan, Nigeria). The −log(*P* value) statistic was extracted for each of the regions containing an insertion. The default cutoff of −log(*P* value) ≥2 was used to define a region having undergone a putative selective sweep.

iHS values (Li et al. 2008) were downloaded from the Prichard Lab data archive. iHS values for regions containing insertions were extracted. |iHS| > 2.5 was assumed to have a higher likelihood of involvement in recent selective sweeps (Li et al. 2008). The sets of insertions associated with selective sweeps using each of the tests were analyzed using GREAT under default settings.

### Phylogenetic Patterns Associate with Gene Insertions

PAML 4.8 (Yang 2007) was run using alignments containing sequences from each of the 6 primate species, for each transcript of the 372 genes identified as containing a human-specific insertion. Alignments were downloaded using the USCS genome browser. The majority of settings and parameters were kept as the defaults from the codonml.ctl file distributed with PAML 4.8. The test was run for the NSsites models 1 (M1a, neutral), 2 (M2a, selection), 7 (M7, beta), and 8 (M8, beta&ω). The log-liklihood values were compared between models 1 and 2 and 7 and 8, and the log ratio test was used to identify genes where the positive selection models (2 and 8) fit the data significantly better than the null models (1 and 7). A Bonferroni correction was applied using the p.adjust function in R 3.0.2.

## Results

Multiple sequence alignments containing human, chimpanzee, gorilla, orangutan, gibbon, and macaque sequences (Karolchik et al. 2014) were searched for regions of human DNA which were deleted in the nonhuman primate comparison species (see Methods). The inclusion of five primate comparison species ensured that the insertions were a true reflection of a recent change in the human lineage rather than an artifact of missing sequence. A total of 283,993 human-specific insertions were identified. About 260,012 insertions which were <10 nucleotides in length were excluded from the analysis as these insertions were assumed to be too short to contain novel regulatory motifs (Bilu and Barkai 2005), and given the difficulty in accurate identification of the correct positions of short indels and therefore the increased difficulty in ensuring correct alignments, it was decided that the study would concentrate only on midlength and long insertions. Of the remaining 23,981 human-specific insertions, 2,712 were found to match known polymorphic insertions in the 1000 genomes project data. Removing these polymorphic insertions left a set of 21,269 insertions, fixed in the human population, having arisen since the most recent common ancestor of humans and chimpanzees. Although the data set was compared against known polymorphic insertions, it is possible that a number of insertions retained in the data set are also polymorphic due to the sparse nature of the indel data in the 1000 genomes project where the power to predict deletions was estimated to be as low as 40% for rare insertions (1000 Genomes Project Consortium 2010). No human-specific insertions were found on the Y chromosome possibly due to the low quality of the assemblies in this region and therefore the low quality of the alignments.

**Fig. 1.—:**
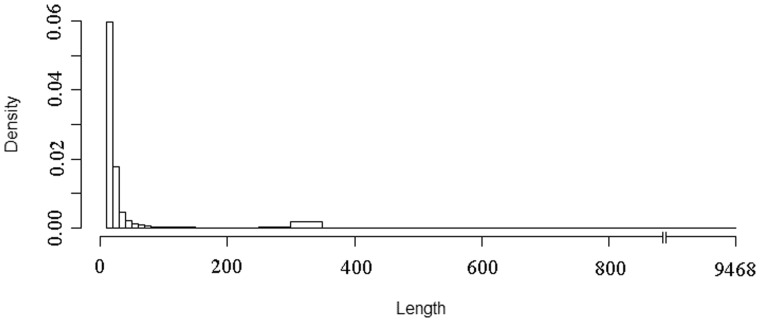
Histogram showing the frequency of human-specific insertions of different lengths.

The insertions ranged in size from 10 to 9,468 nt (fig. 1). Insertions were skewed toward smaller lengths, with over 80% of the human-specific insertions between 10 and 50 nt long. Many of the insertions which were >100 nt corresponded to TE insertions. A noticeable peak occurs in insertions with lengths between 300 and 350 bp, these lengths correspond to the Alu elements. A small peak also occurs for insertions which are approximately 6,000 nt long. These elements correspond to the LINE1 family. Both Alu (Britten 2010) and LINE1 (Beck et al. 2010) elements are known to be actively transposing in humans, albeit in small numbers. These elements would, therefore, be expected to be observed as human-specific insertions more often than the DNA transposons, for example, which have been inert for tens of millions of years.

**Fig. 2.—:**
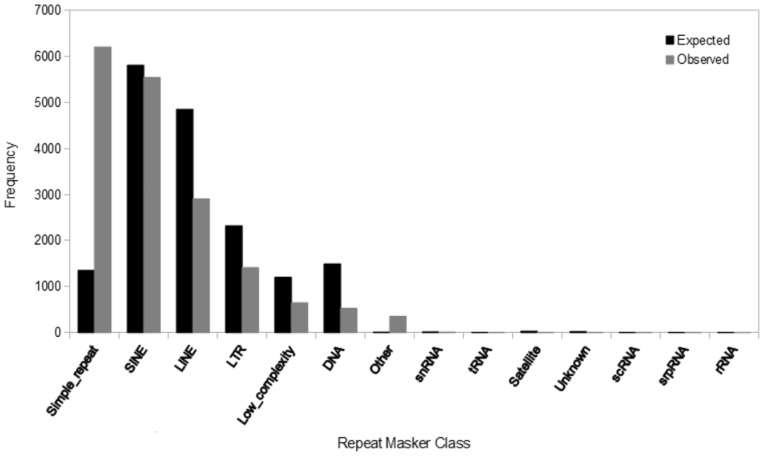
Frequency of insertions corresponding to each RepeatMasker class compared with the expected frequency of elements of each class given the percentage of the human genome consisting of human-specific insertions.

Repeat elements were found to account for at least part of the insertion in 72.6% of cases. Over 93% of these insertions overlapped a repeat element by at least 80%. Although many of these repeats are TEs, simple repeats, low complexity repeats, and RNAs were also included. Simple repeats were observed far more frequently than would be expected given the proportion of RepeatMasker elements that are annotated as simple repeats in the human genome (fig. 2). This may indicate that these elements are frequently created, but eventually lost from the genome. Given that we have removed indels identified in the 1000 genomes project as polymorphic (see Methods), we can assume that these simple repeats have reached fixation in the human genome. This would imply that these particular repeats are not strongly deleterious, despite the evidence that simple repeats even in noncoding regions are often found to be disease causing in humans (Usdin 2008).

The majority of human-specific insertions were found in noncoding regions, 73.8% in nongenic regions. However, 5,582 UCSC genes (Karolchik et al. 2014) were found to contain insertions (this included insertions found in introns, exons, 3′ UTR, and 5′ UTR), 372 of which were found with insertions in exons, 144 in 5′ UTRs, 276 in 3′ UTRs, and the remainder in intronic regions.

### Analysis of Genes Associated with Human-Specific Insertions Shows Enrichment for Neural and Diet-Related Phenotypes

Of the 5,582 genes found with insertions, 4,761 were annotated for use in DAVID (Dennis et al. 2003) and we analyzed these for functional enrichments. Of the 4,761 annotated loci, 2,450 (51%) of the genes were found expressed in brain tissue (*P* = 2.2 × 10^−68^). Subtissues of the brain were found with lower, but still significant, enrichment: The amygdala (*P* = 2.7 × 10^−^^11^), a region of the brain thought to contribute to the processing of memory and emotional reactions (Amunts et al. 2005), and the hippocampus (*P* = 6.7 × 10^−7^), also thought to be involved in memory (Barker and Warburton 2011; Battaglia et al. 2011). The first two most enriched KEGG pathways were focal adhesion and pathways in cancer, neither of which seems an immediately obvious source of differences between humans and nonhuman primates. However, the third most enriched KEGG pathway was the axon guidance pathway (*P* = 2.7 × 10^−10^) which included 67 insertion containing genes out of a total of 73 genes in the pathway. Thus among loci that have gained a human-specific insertion since our common ancestor with chimpanzee, we see a massive enrichment for genes expressed in the brain.

Insertions were identified in exons from 372 genes. The set of genes was enriched for clusters of annotations related to the development of sensory organs, the eye in particular (sensory organ development, *P* = 2.8 × 10^−2^; eye development, *P* = 6.7 × 10^−2^; camera-type eye development, *P* = 8.3 × 10^−2^), and to neuron development (including: Neuron development, *P* = 7.1 × 10^−3^; neuron projection development, *P* = 1.0 × 10^−2^; neuron differentiation, *P* = 2.9 × 10^−2^; axonogenesis, *P* = 5.9 × 10^−2^; axon guidance, *P* = 8.3 × 10^−2^). Other clusters of terms were identified with higher enrichment scores, but these were associated with common housekeeping tasks such as apoptosis or to common molecular functions such as DNA binding. Several tissues were enriched for expression of the genes. Tongue, epithelium, fetal skin, lung, pancreas, lymph, bone marrow, and brain were all enriched at a statistically significant level (table 1). Interestingly, the most statistically significant enriched tissue in this gene set is the tongue (*P* = 1.6 × 10^−2^). The tongue is central to the development of language and while the size and shape of the tongue is conserved between humans and chimpanzees, the musculature has altered considerably to account for changes in the skull shape (Coquerelle et al. 2013).

**Table 1:** DAVID Up-Tissue Results for the 372 Genes with Insertions in Exons

	Percent of Set	*P* Value	Benjamini
Tongue	4.1	1.6 × 10^−2^	7.6 × 10^−1^
Epithelium	16.9	2.2 × 10^−2^	7.2 × 10^−1^
Fetal skin	0.8	3.4 × 10^−2^	7.8 × 10^−1^
Lung	16.1	4.6 × 10^−2^	8.0 × 10^−1^
Pancreas	7.1	5.4 × 10^−2^	8.0 × 10^−1^
Lymph	5.2	6.1 × 10^−2^	7.9 × 10^−1^
Bone marrow	5.5	7.4 × 10^−2^	8.2 × 10^−1^
Brain	42.9	9.6 × 10^−2^	8.6 × 10^−1^

We next used GREAT, a tool designed to predict the function of *cis*-regulatory regions, and we identified 10,095 genes possibly regulated by the regions in which we found human-specific insertions. The analysis identified three disease phenotypes enriched within the data set: Hereditary fructose intolerance syndrome (*P* = 4.39 × 10^−5^), cerebral toxoplasmosis (*P* = 1.14 × 10^−3^), and agoraphobia (*P* = 1.17 × 10^−4^) (supplementary table S1, Supplementary Material online). Human-specific differences in regions which may affect fructose metabolism are interesting, given that the nonhuman ape diet consists of a much higher percentage of fruit than does the human diet (Watts et al. 2012). The cerebral toxoplasmosis association implies a relationship to brain tissue related genes and the agoraphobia association to genes related to behavior and higher brain function, another area where phenotypes differ substantially between humans and nonhuman apes.

The data set was also enriched for a number of mouse phenotypes, particularly phenotypes involved in the development of head, facial, or neural tissues. Tissues involved in teeth and jaw development were particularly prevalent (TS24_upper jaw, tooth, incisor, mesenchyme, dental papilla, *P* = 2.6 × 10^−4^; TS_24_upper jaw, tooth, incisor, epithelium, enamel organ, *P* = 1.1 × 10^−3^). Unexpectedly, the data set was enriched for several timepoints of the development of the vomeronasal organ (TS25_vomeronasal organ: Mesenchyme, *P* = 2.6 × 10^−^^4^; TS21_vomeronasal organ: Epithelium, *P* = 1.1 × 10^−3^; TS21_vomeronasal organ: Epithelium, *P* = 1.1 × 10^−3^), thought to be nonfunctional in humans and apes (Smith et al. 2002). However, the genes identified as involved (*MSX1* and *MSX2*) are also identified in tooth development, an area with known phenotypic differences between humans and apes and hence a much more likely candidate for any function of the insertions than vomeronasal development is.

**Fig. 3.—:**
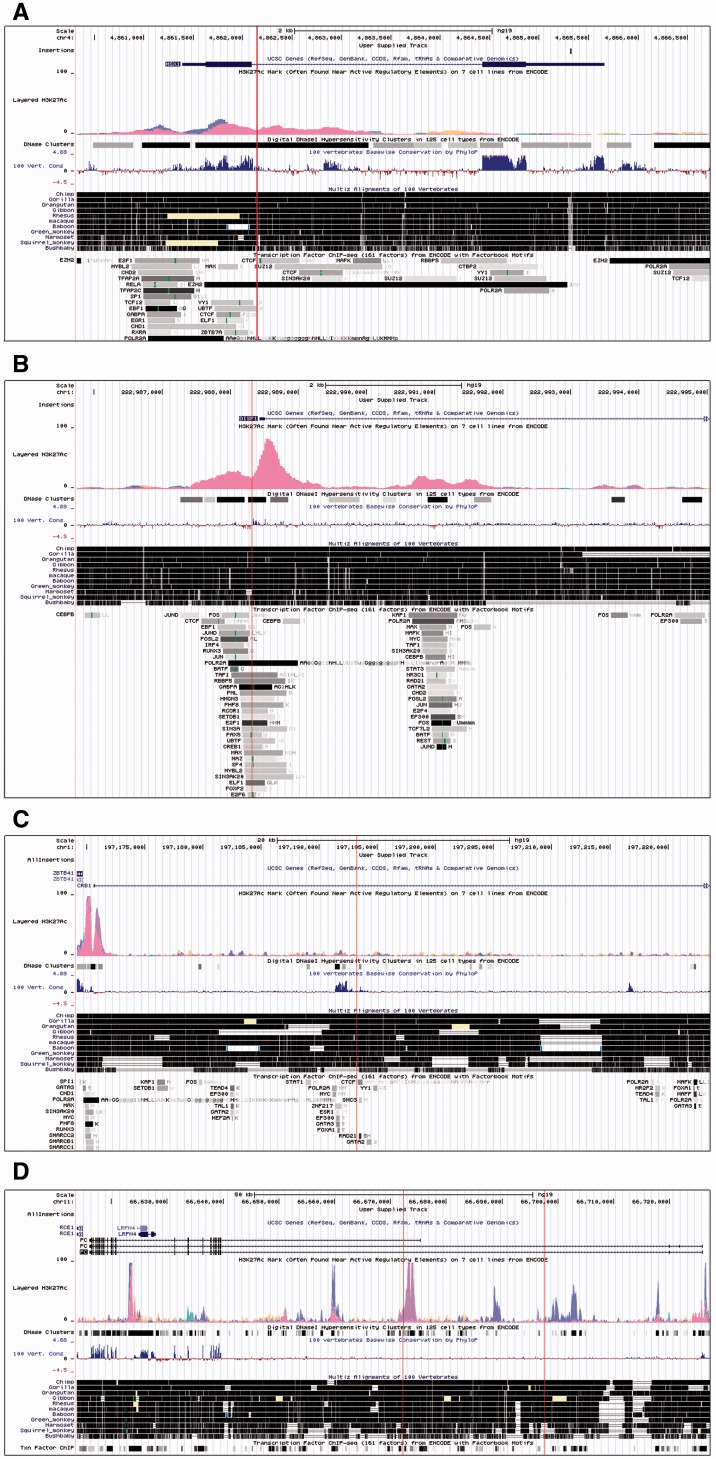
Screenshots from the UCSC Genome Browser (Kent et al. 2002) showing insertions within genes of particular interest in explaining the phenotypic differences between humans and other apes. The red vertical line shows the position of the human-specific insertion. (*A*) MSX1 showing an insertion in the first intron. (*B*) DISP1 showing an insertion in a putative promoter region. (*C*) CRB1 showing an insertion in the first intron. (*D*) *PC* showing two insertions with the same intron, although one is only found in two of the possible transcripts.

A set of strict criteria was used to reduce the number of human-specific insertions to those most likely to affect gene expression. The criteria mandated that insertions overlapped DNAse clusters, regions with a high frequency of TFBS and were found within 5 kb of the gene (see Methods). The resulting set of insertions showed no enrichments related to tissue expression and no known human phenotypic associations. However, one mouse phenotypic association was found: Absent upper incisors (*P* = 1.4 × 10^−7^). Insertions were found in two genes linked to this phenotype: *DISP1* and *MSX1*, a gene associated with Wolf–Hirschhorn syndrome (Nieminen et al. 2003), cleft palate (Vastardis et al. 1996), and oligodontia (Wong et al. 2014) (fig. 3). The *MSX1* insertion falls in the first intron and overlaps a putative *UBTF*-binding motif. *UBTF* is known to activate RNA polymerase I mediated transcription through binding at enhancer regions (Kwon and Green 1994). Enhancer regions have been identified in the intronic regions of several genes (Gardiner et al. 2012; Gillen and Harris 2012) and as such this is an interesting candidate for insertion-mediated expression differences between humans and nonhuman apes.

The subsets of insertions associated with each class of repeat element were also analyzed using GREAT. Several interesting phenotypes were associated with specific classes of repeats (supplementary table S3, Supplementary Material online). SINE insertions were enriched in regions related to neural phenotypes in mouse (abnormal spinal cord dorsal column morphology, *P* = 1.6 × 10^−5^; abnormal neural fold elevation formation, *P* = 1.3 × 10^−^^4^) and to genes showing hindbrain expression (TS18_hindbrain, *P* = 1.1 × 10^−4^; TS21_hindbrain, *P* = 1.1 × 10^−4^). Long terminal repeats (LTRs) were associated with behavioral and memory-related phenotypes in mouse (induced hyperactivity, *P* = 5.0 × 10^−13^; abnormal passive avoidance behavior, *P* = 5.2 × 10^−8^; abnormal avoidance learning behavior, *P* = 3.0 × 10^−7^; abnormal temporal memory, *P* = 1.4 × 10^−6^) and were also associated with several neurally related GO terms (dendrite morphogenesis, *P* = 2.8 × 10^−11^; dendrite development, *P* = 4.7 × 10^−^^10^; forebrain generation of neurons, *P* = 1.2 × 10^−9^; forebrain neuron differentiation, *P* = 1.7 × 10^−9^; positive regulation of neurogenesis, *P* = 5.4 × 10^−6^; pattern recognition receptor signaling pathway, *P* = 1.2 × 10^−4^).

## Insertions Associated with Regions under Positive Selection

We next sought to test whether there were any detectable signatures of selective sweeps associated with human-specific insertions. A priori we believe that we might have little chance of detecting such sweeps, as most of the insertions we are studying are probably old enough that any sweep signal would have decayed, and moreover we have reason to believe that selective sweeps are relatively rare in recent human evolution (Hermandez et al. 2011; Lohmueller et al. 2011). To this end, we used the 1000 Genomes Selection Browser, a database that applies a large number of common scan statistics to the human genome to identify regions under selection in three populations: CEU (Utah residents with ancestry from northern and western Europe), CHB (Han Chinese in Beijing, China), and YRI (Yoruba in Ibadan, Nigeria) (Pybus et al. 2014). The human-specific insertions were compared with the results from a number of these statistics (see Methods). Interestingly, although the insertions were not found to be enriched as a group in regions under selection, we did find several insertions associated with sweeps that may be good candidates for further analysis.

The CLR statistic (Nielson et al. 2005) identified less than 200 insertions in each population (CEU: 167, CHB: 181, YRI: 169), in regions which may have been under selection (−log10 (*P* value)>2) and only a very small number of these are identified in more than one population with no regions containing insertions located in a selective sweep which was identified in all 3 populations. The sets of insertions identified in the CEU and YRI populations showed no enrichment for a particular phenotype; however, the CHB population showed enrichments for genes related to nephroblastoma (*P* = 1.6 × 10^−4^) and hyperopia (*P* = 1.7 × 10^−4^). The insertions related to hyperopia are interesting as these may indicate positive selection related to a change in vision. In particular, an insertion is present in the first intron of the *CRB1* transcript variant 3 (chr1: 197,193,232–197,193,260) (fig. 3*C*). The *CRB1* gene is orthologous to the drosophila crumbs protein, a protein thought to control the development of polarity in the eye.

The Fu’s F (Fu 1997) statistic also identified approximately 200 or fewer insertions for each population (CEU: 201, CHB: 195, YRI: 179), in regions which may have been under selection, there was little overlap between the insertions identified in each population. Both the CHB (supplementary table S4, Supplementary Material online) and CEU (supplementary table S6, Supplementary Material online) populations showed enrichment for terms related to negative regulation of signaling; however, only high-level GO terms were identified. The CHB (supplementary table S5, Supplementary Material online) and YRI (supplementary table S7, Supplementary Material online) populations both showed an enrichment of genes expressed in spinal cord or nervous system tissues.

iHS (Voight et al. 2006) values are useful for detecting ongoing selection. Although the existence of the insertions that we are interested in is assumed to be fixed within the human population, sequence mutations have occurred within these insertions. A search of data from the 1000 genomes project phase 1 found 21,270 SNPs within the human-specific insertions. Exaptation of insertions is often triggered by further mutations and as such it is interesting to see whether our insertions are found in regions currently undergoing selection in humans. Data showing iHS values for regions of the human genome in different populations were downloaded from the Prichard Lab data archive rather than the selection browser as this gave data from a larger number of populations (Coop et al. 2009; Pickrell et al. 2009). No insertion-containing regions were found with an |iHS| >2.5 in all eight populations. However, 52 elements were found for the intersection of the European, Middle Eastern, and South Asian populations. The phenotypes enriched in this data set were almost entirely due to four insertions associated with the pyruvate carboxylase (*PC*) gene, two of which are found in intronic regions (fig. 3*D*). *PC* deficiency or mutations are related to a number of neural phenotypes: Neuronal loss in the cerebral cortex (Lee et al. 2013), periventricular leukomalacia (García-Cazorla et al. 2006), and abnormality of the periventricular white matter (Brun et al. 1999).

### Phylogenetic Patterns Associate with Insertions in Coding Sequences

When an insertion occurs in a protein-coding locus, it is likely that the addition of codons might lead to a cascading response of evolutionary changes within the gene. We decided to characterize patterns of phylogenetic change along the human lineage in those protein-coding genes that have experienced insertions. PAML was used to analyze primate transcript alignments under four different models to determine whether an assumption of positive selection at the codon level fits the genes in which insertions had been identified. The NSsites models 1 (no selection) and 2 (positive selection) were compared, as were the NSsites models 7 (M7, beta) and 8 (M8, beta&ω), where higher log*likelihood scores for models 2 or 8 show a better fit under the assumption of positive selection (supplementary table S8, Supplementary Material online).

Twenty-five genes, out of the 372 tested, showed a significantly better fit to the models under positive selection (LRT; *P* < 0.05). The comparison of models 7 and 8 showed a higher number of transcripts which fit the assumption of positive selection, but there was no difference between the number of genes with at least one transcript fit the assumption. Of these genes, three were associated with known neural phenotypes: *TMCO1*, which is associated with mental retardation (Xin et al. 2010), *TRIOBP* which codes for a protein known to interact with *TRIO*, a protein known to be involved in neural tissue development (O'Brien et al. 2000), and *KIAA0319*, thought to play a role in the development of the cerebral cortex (Darki et al. 2012) and containing mutations linked to dyslexia (Cope et al. 2005; Harold et al. 2006; Pinel et al. 2012; Zhou et al. 2012). Given that the development of language is one of the major factors which differentiate humans from other primates, this is a particularly exciting gene to have identified in the analysis. Three genes were associated with sensory perception (with some overlap to the neural genes): *TAS2R20*, a taste receptor involved in the identification of bitterness (Conte et al. 2002), a phenotype previously identified as being in rapid decline in humans in comparison with other primates (Go et al. 2005); *TRIOBP*, a protein involved in hair cell formation (Kitajiri et al. 2010) and in which mutations have been associated with deafness (Riazuddin et al. 2006; Shahin et al. 2006); and *OR1E2*, a protein associated with smell (Ben-Arie et al. 1994). Genes were also identified which are related to the immune response (*TCF7*, Wu et al. 2012; *IL17RC*, Ho et al. 2010; Hu et al. 2010), a phenotype which has not been identified so far in this analysis, but which is known to differ between humans and nonhuman primates. Although the majority of insertion-containing genes do not appear to show patterns of selection, many of the genes are involved in phenotypes associated with the differences between humans and nonhuman apes.

Given that multiple comparisons increase the likelihood of false positive results, a Bonferroni correction was applied to the LRT *P* values. This reduced the number of genes which fit the assumption of positive selection to 3: *TACC2, TRIOBP*, and *ZNF780B*. The significance of TRIOBP under the more stringent test is a welcome result, given its involvement in tissue development. Although some of the genes thought to be significant under the LRT test without the Bonferroni correction were no longer deemed significant results, they are still of interest given their phenotypes.

## Discussion

A complete understanding of genome evolution would include not only a description of nucleotide turnover along individual lineages but also the insertion and deletion processes that change overall genome content. The recent increase in well-annotated primate genomes and human population studies has provided the resources for an analysis of the human-specific insertions. Our analysis of five primate genomes aligned to humans resulted in the identification of 283,994 such insertions, 23,982 of which were at least 10 nucleotides long. Given that a total of approximately 37,000 deletions were identified in a comparison with the chimpanzee genome (McLean et al. 2011), the number of insertions identified is much larger than expected. This may imply that the insertion of DNA is less detrimental to the host organism, subject to less purifying selection and perhaps greater positive selection. Insertions may, therefore, be a better candidate mechanism to explain the rapid changes in human phenotypes since the most recent common ancestor with chimpanzees than are deletions. The majority of these new insertions were found in noncoding regions and as such, while they may not create direct amino acid changes, they provide a wealth of new genetic information to be utilized in gene regulation.

Many of the phenotypes enriched within the data set were in areas of known variation between humans and nonhuman apes. In particular, neural and cranial-related phenotypes were identified, some of which were identified in protein-coding regions of genes which show phyologenetic patterns associated with positive selection. Enrichment of genes expressed in neural tissues was also shown in an analysis of human-specific deletions (McLean et al. 2011). Given the assumption that many changes related to neural tissues have occurred since the most common ancestor of humans and chimpanzees, leading to the development of advanced cognition and language in humans, it is likely that many different types of mutations have contributed to differences in neural-related phenotypes.

Many of the human-specific insertions identified in this study resulted from recent TE activity. As expected, due to their continued transpositional activity in humans (Huang et al. 2012), SINES were found abundantly. SINE-related insertions were enriched in regions with possible regulatory functions for genes associated with neural phenotypes. Previously, several ancient SINES have been implicated in neural functions (Santangelo et al. 2007; Franchini et al. 2011; Tashiro et al. 2011), and our data, along with other studies (Britten 2010), suggest that there may be more recent SINE-driven neural adaptations. LTR elements were also found to be associated with neural functions; in particular, LTRs were found to be enriched in regions associated with genes related to memory or behavioral phenotypes. It has recently been suggested that other TEs may also play a role in normal human brain function (Guffanti et al. 2013; Hunter and McEwan 2013) as well as having some influence on many psychiatric disorders (Muotri et al. 2009; Baillie et al. 2001; Guffanti et al. 2013).

Very few of the human-specific insertions were found in regions thought to have been under positive selection when analyzed using population genetic methods aimed at detecting the signatures of selective sweeps. Of those that were under positive selection, no clear pattern of phenotypic enrichment was observed. Several insertions in regions associated with positive selection were either found in, or thought to regulate, the *PC* gene. *PC* has been associated with a wide variety of neural functions (Jitrapakdee et al. 2006), one of the most interesting of which may be its role in the biosynthesis of neurotransmitters (Shank et al. 1985). Phenotypes of particular interest which *PC* has been associated with include the following: Neuronal loss in the cerebral cortex (Lee et al. 2013), periventricular leukomalacia (García-Cazorla et al. 2006), and abnormality of the periventricular white matter (Brun et al. 1999). Given the variety of neural functions that *PC* is associated with, and that four insertions were associated with the gene, this may be a very good candidate to analyze further in the search to explain the development of higher neural functions in humans compared with other apes.

Other than neurally related phenotypes, the development of incisors appears to be a particularly likely phenotype for regulation by insertions. In particular, the insertion found in the first intron of *MSX1*, overlapping regulatory binding sites identified in the ENCODE project, would be an interesting candidate for further study. Dentition is known to have changed considerably since the MRCA of humans and chimpanzees. Although the most prominent dental differences between humans and chimpanzees, and indeed other primates, are found in the reduced size of the canines in humans, other differences can also be seen. In particular, the positioning of the lateral incisor is shown to differ significantly in humans compared with nonhuman primates. These differences are possibly a side effect of the reduction in canine size and of the changing skull shape (Fukase 2012). A study involving a family with upper lateral incisor agenesis suggested a link to the known *MSX1* mutation, c.*6C>T. Although a link was seen between the homozygous occurrence of the mutation and the incisor agenesis phenotype, the mutation is a common polymorphism and so additional factors must be involved (Boeira and Echeverrigaray 2012).

Further interesting phenotypes which were identified in several different analyses were those related to sensory development and perception and those which were identified as related to language. *TAS2R20*, *TRIOBP*, and *OR1E2* were all identified as sensory-related genes which contained insertions and which showed phylogenetic patterns of positive selection. *KIAA0319* also showed signatures of positive selection and is known to be related to dyslexia in individuals with mutations. It is therefore possible that this insertion may have had a role to play in the development of language.

Our analysis suggests that human-specific insertions have played a large role in the recent evolution of humans. Although many of the insertions are likely to have been fitness neutral or nearly so, a much smaller number may be assumed to have had a substantial effect on the differentiation of humans from other apes. However, the large number identified precludes a detailed functional examination of each insertion. As more data are obtained on the functionality of regulatory motifs and regions and on areas of the human genome which have been under recent selection, further scans may be able to reduce the large number of insertions possibly related to recently evolved human-specific functionality.

## Supplementary Material

Supplementary tables S1–S8 are available at *Genome Biology and Evolution* online (http://www.gbe.oxfordjournals.org/).

Supplementary Data
