# Supplementary material for: The Role of DNA Insertions in Phenotypic Differentiation between Humans and Other Primates
Source: Genome Biol Evol. 2015 Jan 28;7(4):1168–78. doi: 10.1093/gbe/evv012 (PMC4419785; doi:10.1093/gbe/evv012)
Supplement: Supplementary Data [file supp_7_4_1168__index.html]

The Role of DNA Insertions in Phenotypic Differentiation between Humans and Other Primates — Supplementary Data 

# The Role of DNA Insertions in Phenotypic Differentiation between Humans and Other Primates

## Supplementary Data

files

**Files in this Data Supplement:**

- Supplementary Data - docx file
