# Supplementary material for: The Role of DNA Insertions in Phenotypic Differentiation between Humans and Other Primates
Source: Genome Biol Evol. 2015 Jan 28;7(4):1168–78. doi: 10.1093/gbe/evv012 (PMC4419785; doi:10.1093/gbe/evv012)
Supplement: Supplementary Data [file supp_evv012_Supplimentary_tables.docx]

| **Phenotype** | **Gene** | **Insertion** |
| --- | --- | --- |
| Hereditary Fructose Intolerance Syndrom | *ALDOB* | -44,578, -30,493, -26,261, -26,132, -24,480, -12,060 |
| Agoraphobia | *ADORA2A* | -110,159, -25,433 |
| Agoraphobia | *CAMKK2* | +78,444 |
| Agoraphobia | *HTR1A* | -176,424, -90,865, +18,436, +53,982, +136,985, +137,030, +137,058, +248,816, +421,562, +429,495, +608,809, +632,231, +778,742, +781,330, +786,626, +831,726, +877,051, +911,499, +936,862, +961,970 |
| Agoraphobia | *P2RX7* | -43,115, -23,418, +30,789, +66,788 |
| Agoraphobia | *SLC6A2* | +20,836, +51,928, +71,925 |
| Agoraphobia | *SPECC1L* | +46,581, +131,307 |
| Cerebral Toxoplasmosis | *IL6ST* | -798,764, -790,023, -739,008, -723,370, -679,872, -559,729, -547,952, -441,413, -402,638, -377,698, -263,578, -236,676, -165,574, -56,780, -19,971, +72,177, +123,013 |

Table S1. GREAT Disease Ontology results for the full set of insertions

| **MGI expression** | **Binom Raw P value** | **Binom FDR Q-val** | **Number of Genes** |
| --- | --- | --- | --- |
| TS28_forestomach | 2.2e-5 | 1.0e-3 | 3 |
| TS24_palate;epithelium | 6.6e-5 | 2.6e-3 | 2 |
| TS21_vomeronasal organ; mesenchyme | 2.6e-4 | 7.9e-3 | 2 |
| TS18_4^th^ arch; mesenchyme; mesenchyme derived from neural crest | 2.6e-4 | 7.9e-3 | 2 |
| TS24_upper jaw; tooth; incisor; mesenchyme; dental papilla | 2.6e-4 | 7.9e-3 | 2 |
| TS25_vomeronasal organ; mesenchyme | 2.6e-4 | 7.9e-3 | 2 |
| TS18_3rd arch; mesenchyme; mesenchyme dervived from neural crest | 2.6e-4 | 7.9e-3 | 2 |
| TS15_future rhombencephalon; roof plate | 6.0e-4 | 1.4e-2 | 3 |
| TS17_diencephalon; roof plate | 6.0e-4 | 1.4e-2 | 4 |
| TS28_stomach mucosa | 6.7e-4 | 1.5e-2 | 5 |
| TS15_rhombomere 05; roof plate | 1.1e-3 | 2.1e-2 | 1 |
| TS24_upper jaw; tooth; incisor; epithelium; enamel organ | 1.1e-3 | 2.1e-2 | 1 |
| TS21_vomeronasal organ; epithelium | 1.1e-3 | 2.1e-2 | 1 |
| TS25_vomeronasal organ; epithelium | 1.1e-3 | 2.1e-2 | 1 |
| TS26_central nercous system; nerve; cranial; vestibulocochlear VIII; cochlear component | 1.7e-3 | 2.8e-2 | 2 |
| TS23_tail; skeleton; axial skeleton; vertebral cartilage condensation | 2.3e-3 | 3.4e-2 | 2 |
| TS24_stomach; glandular region; epithelium | 3.0e-3 | 4.1e-2 | 1 |
| TS25_limb; skeleton | 3.0e-3 | 4.1e-2 | 3 |

Table S2. MGI Expression enrichments for the total set of insertions.

| **Repeat Class** | **# insertions** | **Mouse phenotype** | **GO: Biological Process** | **MGI Expression: detected** |
| --- | --- | --- | --- | --- |
| Simple_repeat | 6211 | audiogenic seizures (p=2.3e-5) | response to low-density lipoprotein particle stimulus (p=2.7e-4) | TS14_urogenital system (p=1.5e-4)  TS15_bulbus cordis; rostral half (p=6.4e-4) |
| SINE | 5546 | abnormal spinal cord dorsal column morphology (p=1.6e-5),  abnormal neural fold elevation formation (p=1.3e-4) |  | TS18_hindbrain; floor plate (p=1.1e-4), TS21_hindbrain; floor plate (p=1.1e-4) |
| LINE | 2907 | decreased lean body mass (p=2.9e-5) | Gamma-aminobutyric signalling pathway (p=6.2e-5)  serotonin receptor signalling pathway (p=1.1e-4) |  |
| LTR | 1411 | induced hyperactivity (p=5.0e-13),  abnormal passive avoidance behavior (p=5.2e-8),  abnormal avoidance learning behavior (p=3.0e-7),  abnormal temporal memory (p=1.4e-6),  abnormal excitatory postsynaptic potential (p=3.9e-5) | dendrite morphogenesis (p=2.8e-11)  dendrite development (p=4.7e-10)  forebrain generation of neurons (p=1.2e-9)  forebrain neuron differentiation (p=1.7e-9)  positive regulation of neurogenesis (p=5.4e-6)  pattern recognition receptor signaling pathway (p=1.2e-4)  innate immune response-activating signal transduction (p=1.3e-4)  negative regulation of adenylate cyclase activity (p=1.8e-4)  negative regulation of lyase activity (p=1.9e-4)  renal vesicle development (p=2.0e-4)  cellular response to prostaglandin stimulus (p=3.8e-4) |  |
| Low_complexity | 648 |  |  |  |
| DNA | 528 |  |  |  |
| Other | 354 |  |  | TS17_forelimb bud; ectoderm; apical ectodermal ridge (p=7.9e-5) |
| snRNA | 12 |  |  |  |
| tRNA | 4 |  |  |  |
| Satellite | 3 |  |  |  |
| Unknown | 3 |  |  |  |
| scRNA | 2 |  |  |  |
| srpRNA | 2 |  |  |  |
| rRNA | 1 |  |  |  |

Table S3. Phenotypes enriched in set of insertions overlapping repeat motifs

| **Go term name** | **Binom Raw P value** | **Binom FDR Q-val** | **Number of Genes** |
| --- | --- | --- | --- |
| Negatve regulation of signal transduction | 6.6e-11 | 5.8e-7 | 32 |
| Negative regulation of cell communication | 2.7e-10 | 7.9e-7 | 33 |
| Negative regulation of signaling | 2.8e-10 | 6.1e-7 | 33 |
| Regulation of signal transduction | 5.8e-10 | 8.5e-7 | 65 |
| Regulation of cell communication | 4.2e-9 | 4.1e-6 | 55 |
| Negative regulation of response to stimulus | 9.0e-9 | 7.9e-6 | 33 |
| Negative regulation of cellular biosynthetic process | 2.5e-8 | 2.0e-5 | 44 |
| Symbiosis, encompassing mutualism through parasitism | 7.1e-8 | 4.8e-5 | 9 |
| Negative regulation of cellular metabolic process | 1.2e-7 | 7.7e-5 | 49 |
| Negative regulation of nucleobase-containing compound metabolic process | 1.5e-7 | 9.1e-5 | 41 |
| Negative regulation of nitrogen compound metabolic process | 1.6e-7 | 9.0e-5 | 41 |
| Negative regulation of macromolecule biosynthetic process | 2.3e-7 | 1.2e-4 | 42 |
| Negative regulation of cellular macromolecule biosynthetic process | 2.8e-7 | 1.4e-4 | 41 |
| Interaction with host | 2.9e-7 | 1.4e-4 | 7 |
| Negative regulation of metabolic process | 3.2e-7 | 1.4e-4 | 50 |
| Negative regulation of metabolic process | 3.2e-7 | 1.4e-4 | 50 |
| Negative regulation of macromolecule metabolic process | 4.2e-6 | 1.7e-3 | 49 |
| Negative regulation of RNA metabolic process | 4.0e-6 | 1.3e-3 | 38 |
| Negative regulation of transcription from RNA polymerase II promoter | 5.2e-6 | 1.5e-3 | 25 |
| Negative regulation of transcription, DNA dependent | 6.9e-6 | 1.8e-3 | 37 |

Table S4. Go term enrichments for the set of insertions predicted by Fu's F statistic to be under positive selection in the CHB population.

| **MGI expression** | **Binom Raw P value** | **Binom FDR Q-val** | **Number of Genes** |
| --- | --- | --- | --- |
| TS22_Ovary | 1.7e-6 | 1.4e-2 | 18 |
| TS22_reproductive system; female | 1.9e-6 | 8.0e-3 | 18 |
| TS16_future spinal cord ; neural tube | 2.3e-6 | 6.5e-3 | 8 |
| TS22_reproductive system; male | 4.3e-6 | 9.1e-3 | 21 |
| TS11_node | 9.0e-6 | 9.4e-3 | 8 |
| TS23_axial skeleton | 2.0e-5 | 1.5e-2 | 29 |
| TS15_1st arch; mandibular component; mesenchyme | 2.7e-5 | 1.9e-2 | 4 |
| TS23_axial skeleton; thoracic region | 2.9e-5 | 2.6e-2 | 22 |
| TS23_sternum | 3.5e-5 | 2.1e-2 | 9 |
| TS20_genital tubercule | 4.6e-5 | 2.4e-2 | 9 |
| TS16_limb | 6.2e-5 | 2.7e-2 | 8 |
| TS26_metanephros | 8.2e-5 | 3.1e-2 | 18 |
| TS15_1st arch; mandibular component | 1.1e-4 | 3.2e-2 | 6 |
| TS23_arytemoid | 1.3e-4 | 3.5e-2 | 3 |
| TS26_renal/urinary system | 1.3e-4 | 3.3e-2 | 18 |
| TS24_renal/urinary system | 1.3e-4 | 3.3e-2 | 15 |
| TS17_urogenital system associated mesenchyme | 1.4e-4 | 3.5e-2 | 10 |
| TS23_embryo; mesenchyme | 1.5e-4 | 3.5e-2 | 21 |
| TS19_main bronchus | 1.7e-4 | 3.6e-2 | 4 |
| TS28_hippocampus layer; granule cell layer | 1.8e-4 | 3.9e-2 | 3 |

Table S5. MGI Expression enrichments for the set of insertions predicted by Fu's F statistic to be under positive selection in the CHB population.

| **Go term name** | **Binom Raw P value** | **Binom FDR Q-val** | **Number of Genes** |
| --- | --- | --- | --- |
| Negative regulation of signal transduction | 8.5e-8 | 7.5e-4 | 23 |
| Negative regulation of cell communication | 2.5e-7 | 1.1e-3 | 24 |
| Negative regulation of signaling | 2.6e-7 | 7.5e-4 | 24 |
| Regulation of glomerulus development | 3.0e-6 | 6.5e-3 | 4 |
| Negative regulation of response to stimulus | 4.1e-6 | 7.2e-3 | 24 |
| Cartilage development | 5.5e-5 | 3.5e-2 | 9 |

Table S6. Go term enrichments for the set of insertions predicted by Fu's F statistic to be under positive selection in the CEU population.

| **MGI expression** | **Binom Raw P value** | **Binom FDR Q-val** | **Number of Genes** |
| --- | --- | --- | --- |
| TS26_central nervous system; nerve; cranial; vestibulocochlear VIII; cochlear component | 1.0e-10 | 2.8e-5 | 2 |
| TS12_future spinal cord; neural tube | 7.9e-6 | 1.6e-2 | 5 |
| Theiler_stage_4 | 1.0e-5 | 1.4e-2 | 36 |
| TS3_8-cell stage | 3.6e-5 | 3.8e-2 | 27 |
| TS4_inner cell mass | 6.1e-5 | 4.7e-2 | 32 |

Table S7. MGI Expression enrichments for the set of insertions predicted by Fu's F statistic to be under positive selection in the YRI population.

|  | **Symbol** | **Chimp** | **Gorilla** | **Orangutan** | **Gibbon** | **Macaque** | **p(1vs2)** | **p(7vs8)** |
| --- | --- | --- | --- | --- | --- | --- | --- | --- |
| uc001avg.3 | PRDM2 | 1.001 | 1.738 | 0.696 | 2.250 | 1.123 | 4.0e-3 | 4.1e-3 |
| uc001gdj.5 | TMCO1 | 1.417 | -0.314 | -0.0474 | -0.441 | **-2.296** | 3.3e-2 | 1.7e-2 |
| uc001hsn.4 | OBSCN | **-5.325** | **-7.875** | **-12.325** | **-13.245** | **-17.471** | 1.5e-4 | 1.4e-4 |
| uc001hsq.2 | TMCO1 | **-5.983** | **-8.755** | **-14.176** | **-15.209** | **-19.294** | 9.8e-4 | 4.5e-4 |
| uc001lfv.3 | TACC2 | **-2.657** | -1.979 | **-4.580** | **-4.999** | **-7.237** | 4.2e-5 | 3.2e-5 |
| uc001llw.3 | GPR123 | -0.783 | 0.990 | **-4.138** | **-3.198** | **-4.695** | 2.7e-2 | 2.5e-2 |
| uc001oyf.3 | GDPD4 | 2.654 | 0.304 | 0.274 | 0.339 | 0.004 | 3.9e-2 | 3.9e-2 |
| uc001qzm.2 | TAS2R20 | 0.168 | -0.746 | -0.969 | -0.563 | -0.409 | 1.1e-2 | 5.1e-3 |
| uc001vge.3 | NEK5 | -1.577 | -1.762 | **-2.760** | **-3.351** | **-3.159** | 2.3e-2 | 2.3e-2 |
| uc002ble.3 | ALPK3 | **-2.796** | -1.925 | **-3.291** | **-4.254** | **-6.019** | 3.3e-4 | 3.4e-4 |
| uc002fyr.4 | CXCL16 | 0.454 | -1.905 | -1.188 | -1.290 | 1.351 | 5.7e-3 | 5.7e-3 |
| uc002jxz.4 | SGSH | -0.378 | **-2.250** | -1.719 | **-2.852** | **-4.059** | 2.4e-2 | 1.8e-2 |
| uc002jya.4 | SGSH | 0.766 | -1.642 | -0.909 | **-2.283** | **-3.351** | 1.6e-2 | 1.6e-2 |
| uc002omu.3 | ZNF780B | **-2.298** | -1.950 | **-3.598** | -1.784 | **-3.238** | 6.1e-5 | 2.7e-5 |
| uc002omv.3 | ZNF780B | **-2.226** | -1.942 | **-2.963** | -1.517 | **-2.676** | 2.9e-4 | 2.9e-4 |
| uc002ybj.1 | C20orf197 | -0.677 | -0.374 | -0.339 | 0.398 | 0.757 | 5.0e-3 | 5.0e-3 |
| uc003atr.3 | TRIOBP | -0.589 | 0.011 | **-3.357** | **-2.790** | **-4.556** | 1.0e-6 | 8.2e-7 |
| uc003atu.3 | TRIOBP | -0.453 | 0.165 | **-3.132** | **-2.617** | **-4.338** | 5.6e-7 | 3.1e-7 |
| uc003hxk.3 | TET2 | -0.517 | **-2.546** | **-2.635** | -1.693 | **-3.864** | 3.0e-3 | 3.0e-3 |
| uc003kyt.3 | TCF7 | -1.421 | -1.533 | **-2.098** | **-2.184** | **-2.959** | 4.9e-2 | 4.6e-2 |
| uc003kyu.2 | TCF7 | -1.539 | -1.539 | **-2.192** | -1.944 | **-2.867** | 4.8e-2 | 4.8e-2 |
| uc003kyv.3 | TCF7 | -1.422 | -1.171 | **-2.104** | -1.494 | **-2.788** | 4.6e-2 | 4.2e-2 |
| uc003kza.3 | TCF7 | -1.422 | -1.170 | **-2.104** | -1.664 | **-2.790** | 4.0e-2 | 3.1e-2 |
| uc004dcx.2 | DMD | **-3.318** | -1.571 | **-5.154** | **-6.181** | **-7.537** | 4.6e-2 | 2.2e-2 |
| uc004emn.3 | TBC1D8B | -1.358 | **-2.544** | **-2.323** | **-3.062** | **-3.128** | 3.6e-2 | 3.5e-2 |
| uc004emo.3 | TBC1D8B | **-2.196** | **-3.369** | **-2.967** | **-4.279** | **-4.670** | 3.8e-2 | 3.5e-2 |
| uc009xez.2 | OBSCN | **-5.814** | **-8.597** | **-13.933** | **-14.623** | **-18.352** | 1.9e-3 | 1.6e-3 |
| uc009xzx.3 | TACC2 | **-2.871** | **-2.150** | **-4.608** | **-5.031** | **-7.251** | 8.1e-5 | 6.7e-5 |
| uc009zfj.2 | C12orf57 | -0.625 | 0.298 | 0.423 | 0.942 | 1.251 | 8.0e-3 | 8.0e-3 |
| uc010hcv.3 | IL17RC | -1.934 | -0.922 | **-1.987** | -1.381 | **-3.994** | 4.5e-2 | 3.6e-2 |
| uc010qtv.2 | TACC2 | **-2.778** | **-2.106** | **-4.547** | **-4.936** | **-7.248** | 1.5e-4 | 8.7e-5 |
| uc010tqj.3 | BC068095 | 1.742 | 2.258 | 0.134 | 0.776 | -1.053 | 4.0e-2 | 3.7e-2 |
| uc010vre.2 | OR1E2 | -1.460 | -1.321 | -1.204 | -0.181 | -1.943 | 7.2e-3 | 6.7e-3 |
| uc010xsi.2 | HPN-AS1 | -0.157 | 1.755 | -0.917 | **-2.017** | -1.051 | 4.2e-2 | 3.9e-2 |
| uc011atq.2 | IL17RC | **-2.181** | -1.099 | -1.789 | -1.286 | **-3.751** | 2.3e-2 | 2.0e-2 |
| uc011cez.2 | TET2 | -0.518 | **-2.547** | **-2.636** | -1.695 | **-3.865** | 2.8e-3 | 2.7e-3 |
| uc011djp.2 | KIAA0319 | -1.340 | **-2.430** | **-2.183** | -1.925 | **-5.065** | 4.8e-2 | 4.8e-2 |
| uc021pvr.1 | AX747977 | 2.251 | -0.035 | -0.869 | -0.303 | -0.283 | 1.7e-2 | 1.7e-2 |
| uc031prd.1 | TMCO1 | 1.417 | 1.417 | -0.025 | 0.264 | -1.588 | 1.9e-2 | 1.9e-2 |

Table S8. dN-dS values calculated for each branch in the primate alignment using the Z-test of evolution (Mega 6) are shown for transcripts containing human specific insertions. Significant values are shown in bold. P values for the PAML comparison of nssites models 1 (no selection) and 2 (positive selection) and for the PAML comparison of nssites models 7 (M7, beta) and 8 (M8, beta&ω).
